# Predominance of CTX-M-15 among ESBL Producers from Environment and Fish Gut from the Shores of Lake Victoria in Mwanza, Tanzania

**DOI:** 10.3389/fmicb.2016.01862

**Published:** 2016-12-01

**Authors:** Nyambura Moremi, Elizabeth V. Manda, Linda Falgenhauer, Hiren Ghosh, Can Imirzalioglu, Mecky Matee, Trinad Chakraborty, Stephen E. Mshana

**Affiliations:** ^1^Department of Microbiology and Immunology, Weill Bugando School of MedicineMwanza, Tanzania; ^2^Institute of Medical Microbiology, Justus Liebig UniversityGiessen, Germany; ^3^German Center for Infection Research, Partner site Giessen-Marburg-Langen, Campus GiessenGiessen, Germany; ^4^Department of Microbiology/Immunology, Muhimbili University of Health and Allied SciencesDar es Salaam, Tanzania

**Keywords:** *bla*_CTX-M-15_, fish, environment

## Abstract

Extended-Spectrum Beta-Lactamase (ESBL)-producing bacteria are a common cause of healthcare and community-associated infections worldwide. The distribution of such isolates in the environment and their presence in fish as a result of sewage contamination is not well-studied. Here we examined fish and environmental samples from Mwanza city for the presence of ESBL-producing bacteria. From 196 fish sampled from local markets, 26 (13.3%) contained lactose-fermenting ESBL-producing bacteria, while 39/73 (53.4%) environmental samples from the same area were ESBL producers. Antibiotic resistance genes, multi locus sequence types (MLST) and plasmid replicon types in 24 selected isolates from both populations were identified with whole genome sequencing using Illumina MiSeq. Nine of eleven sequenced fish isolates had the *bla*_CTX-M-15_ gene whereas 12/13 from environment carried *bla*_CTX-M-15_. Antibiotic resistance genes encoding resistance to sulfonamides (*sul1/sul2*), tetracyclines [*tet(A)/tet(B)*] fluoroquinolones [e.g., *aac(6*′*)-Ib-c*r, *qnr*S1], aminoglycosides [e.g., *aac(3)-lld, str*B, *str*A,] and trimethoprim (e.g., *dfr*A14) were detected. *E. coli* sequence type ST-38 (2) and ST-5173 (2) were detected in isolates both from the environment and fish. IncY plasmids carrying *bla*_CTX-M-15_, *qnrS*1, *str*A, and *str*B were detected in five environmental *E. coli* isolates and in one *E. coli* isolate from fish. Our data indicate spillage of resistant environmental isolates into Lake Victoria through the sewage system. Persistence of *bla*_CTX-M-15_ in the Mwanza city environment is complex, and involves both clonal spread of resistant strains as well as dissemination by commonly occurring IncY plasmids circulating in isolates present in humans, the environment as well as in the food chain.

## Introduction

The extended-spectrum beta-lactamases (ESBL)-producing Gram-negative bacteria *Escherichia coli* and *Klebsiella* spp., particularly those producing CTX-M enzymes, have emerged as important causative agents of healthcare-associated infections across the world (Hawkey and Jones, 2009). Apart from being present in hospitals and clinics, ESBL-producing *E. coli* strains are prevalent in the community and are reported to be responsible for community-acquired bacterial infections (Arpin et al., 2005; Pitout et al., 2005). Studies from different areas of the world show an association between the presence of ESBL-producing *E. coli* and exposure to either food or contaminated water (Ho et al., 2011; Laube et al., 2013; Xi et al., 2015). The presence of ESBL-producing *E. coli* in the community has led to the hypothesis that there could be a transmission of these strains from human waste to the environment (Martinez, 2009a). Evidence in the literature has also documented the probable horizontal transfer of resistance genes from either human sewage or clinical isolates to fish in rivers or lakes in which drainage of wastewater from treatment plants occurs (Kümmerer, 2009; Martinez, 2009b; Jiang et al., 2012; Blaak et al., 2014). Multiple genotypes of ESBL-producing *E. coli* have been found in animals and humans in Mwanza (Mshana et al., 2011, 2016; Seni et al., 2016). There are few studies from developed countries on the presence of ESBL-producing isolates in fish and environment samples and the role played by the food chain in transmission of resistance genes through contamination by human and animals sewage and anthropogenic activities in relation to water bodies (Zurfluh et al., 2013; Abgottspon et al., 2014). In Mwanza, a port city with a population of 750,000 located on the southern shores of Lake Victoria (Fitzpatrick et al., 2015), effluents from wastewater treatment plants and pit-latrines used by most of households located in the hills drain into the lake, which is also a source of fish consumed by residents as staple food. We previously used conventional phenotypic characterization and whole genome sequencing to examine samples obtained from hospitals, from rural farming communities including animals, farmers and households, in Mwanza for the presence of ESBL-producing *Enterobacteriaceae*. Our data indicate clonal spread of bacteria belonging to a small number of STs present in all populations investigated, but also suggest that commonly occurring promiscuous plasmids are involved in resistance dissemination (Fortini et al., 2015; Mshana et al., 2016). Here we examined for the presence of ESBL-producing bacteria in fish obtained from Lake Victoria as well as environmental samples obtained from the city. Our results indicate that *E. coli* genotypes that were observed in humans and animals before are also present in environment and Fish.

## Materials and methods

### Isolation and identification of bacterial isolates

Ten fish markets located in different urban and rural sections of the Mwanza region were randomly selected for the study. These markets receive fishes obtained from different fishing sites within Lake Victoria. A total of 196 Nile tilapia (*Oreochromis niloticus*) fish from randomly picked vendors were sampled with an average of 15 to 20 fish at each market between July and September 2015. Fish were first washed with saline, following which a sterile surgical blade was used to open the carcass and to make a longitudinal incision along the gut. The incision was opened and the gut contents were swabbed using a sterile swab.

**Figure 1:**
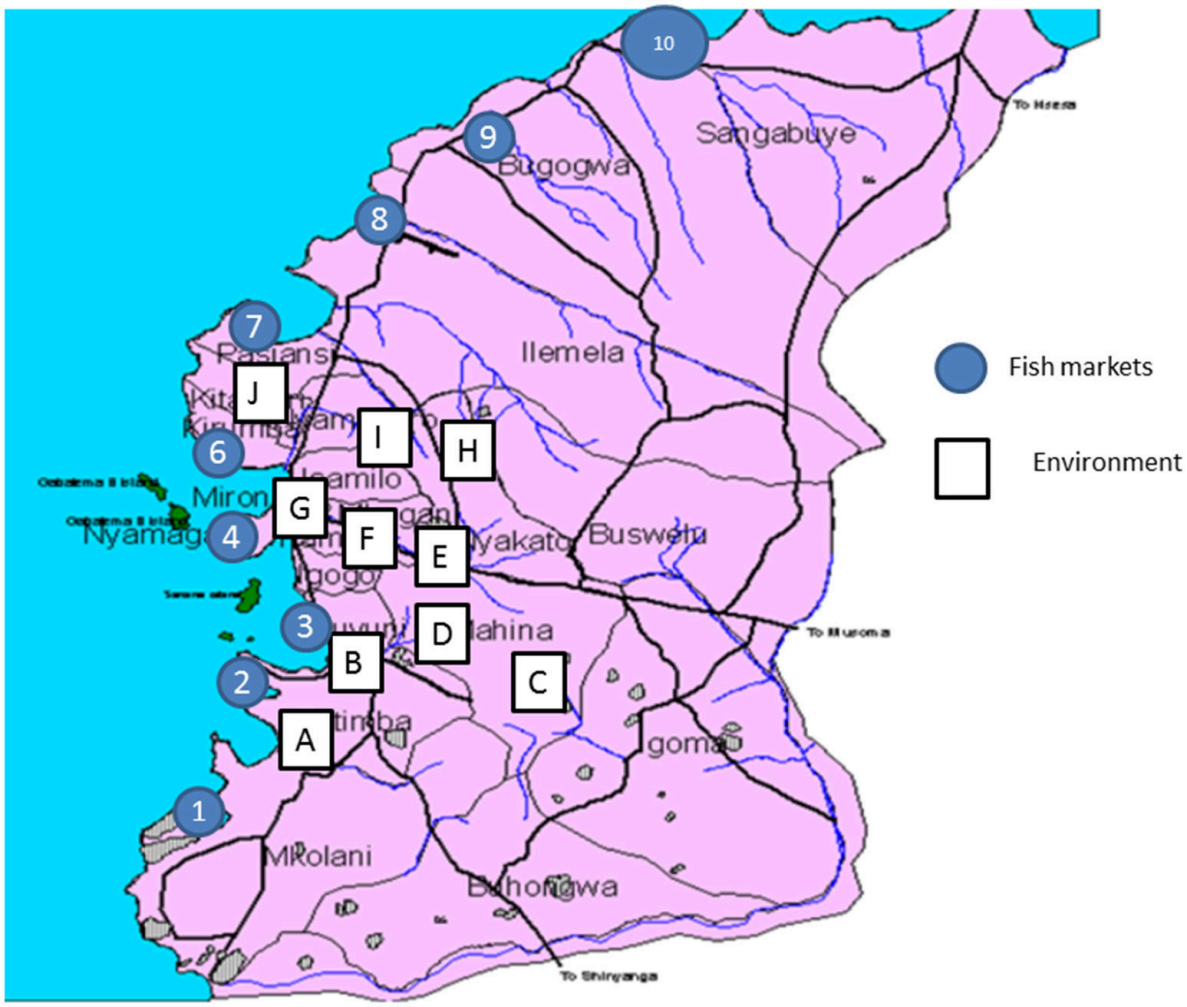
**Map of Mwanza City showing fish markets and sites from which environmental samples were obtained**.

In addition, at ten sites including the Ngerengere River that crosses the city, drain waste and possible sewage from households located in the surrounding hills were sampled (environmental samples). Environmental samples included dirty muddy water samples from different location in the city (Figure 1). For each site between six to seven samples were taken from different locations (Figure 1). About 3 ml of each sample was collected using sterile 25 ml falcon tube (BD, Nairobi, Kenya). A sample was mixed with sterile 0.9% saline at ratio of 1:1 and vortexed to produce a homogenous solution.

Using swabs, MacConkey agar (Oxoid, Basingstoke, UK) plates supplemented with 2 mg/L cefotaxime (Medochemie Ltd, Limassol, Cyprus) were inoculated to screen for ESBL-producing *Enterobacteriaceae*. Enteric bacilli were identified using colony morphology and differentiated based on lactose fermentation on MacConkey agar. Single colonies from predominant lactose fermenting bacteria were picked for further identification using several biochemical tests (Triple Sugar Iron Agar, Simmons' citrate Agar, Sulfur-Indole-Motility test and Urease test) (Murray et al., 1995). In case of ambiguous results, the VITEK® 2 system (BioMérieux, Marcy l'Etoile, France) was used to confirm identification. A confirmed ESBL-producing *E. coli* isolate was used as a positive and *E. coli* ATCC 25922 as a negative control. ESBL isolates were stored as glycerol cultures at −80°C and used for further characterization.

### Antimicrobial susceptibility testing

Antimicrobial susceptibility testing was done by disk diffusion method as recommended by the Clinical Laboratory Standards Institute (CLSI) guidelines (Wayne, 2012). A bacterial suspension of 0.50 McFarland standard turbidity was prepared from pure culture. An inoculum was then plated on Mueller-Hinton Agar plates (HiMedia, Mumbai, India) and the following antibiotic disks were set: Tetracycline (2 μg), ciprofloxacin (5 μg), gentamicin (10 μg), or trimethoprim/sulphamethoxazole (1.25/23.75 μg) (Oxoid, Hampshire, UK). The plates were incubated aerobically at 37°C for 18–24h. The diameters of the respective zone of inhibitions were measured and interpreted following CLSI 2012 guidelines (Wayne, 2012). Disk approximation method based on CLSI guidelines was used to confirm ESBL production and for selected isolates ESBL production was further identified using VITEK® 2 system (BioMérieux, Marcy l'Etoile, France) and in addition the MIC for cefepime, carbapenems and colistin were determined in all selected isolates.

Using STATA version 11 the two-sample test of proportion was done to compare the rates of resistance between ESBL isolates from fish and those from environment. A *p*-value < 0.05 was used to indicate a significant difference at 95% confidence interval.

### Whole genome sequencing

Eleven ESBL-producing isolates obtained from fish and 13 *E. coli* from the environmental samples were chosen for whole genome sequencing (WGS). DNA was isolated using the Purelink Genome DNA Mini kit (Invitrogen, Darmstadt, Germany) according to the manufacturer's instruction. WGS was carried out using an Illumina Nextera XT library with 2x300bp paired-end reads on an Illumina MiSeq instrument (Illumina, San Diego, CA, USA). The raw data was assembled using SPAdes (version 3.0) (Bankevich et al., 2012). Contigs from *E. coli* isolates were ordered by using MAUVE (Rissman et al., 2009) and *E. coli* MG1655 (accession number U00096.3) were chosen as a reference for all *E. coli* isolates, *Citrobacter freundii* strain P10159 for *Citrobacter braakii* isolates (accession number CP012554; no complete genome available for *C. braakii*), *Klebsiella pneumoniae* strain ATCC BAA-2146 (accession number CP006659) for *K. pneumoniae* and *Enterobacter cloacae* strain 34977 (accession number CP010376) for *E. cloacae* isolates. Pseudogenomes were created and whole genome phylogenetic analysis was subsequently performed by using ParSNP package of Harvest Suite (Treangen et al., 2014). The raw sequencing data of the sequenced isolates are available at the European Nucleotide Archive (ENA) under the project number PRJEB12361.

### *In silico* analyses of resistance genes, MLST, plasmid replicon types, and quinolone resistance-determining regions (QRDR) mutations

Sequences were analyzed for their multi locus sequence types, transferrable resistance genes, plasmid replicon types and pMLST using MLST 1.8, ResFinder, Plasmidfinder and pMLST software from the Center for Genomic Epidemiology (Larsen et al., 2012; Zankari et al., 2012; Carattoli et al., 2014). Search for plasmid-encoded heavy metal resistances and detergence resistance was performed using blastn with the references given in Supplementary Table 1.

The location of *bla*_CTX-M-15_ was determined by analyzing the contigs harboring *bla*_CTX-M-15_ using blastn. The whole genome sequences were compared with the plasmid pPGRT46 (accession number KM023153.1) using BRIG and blastn (Alikhan et al., 2011; Fortini et al., 2015). Quinolone resistance-determining regions (QRDR) mutations were identified by *silico* analysis by comparing the sequence using a reference sequence from a quinolone-susceptible *Enterobacteriaceae* strain (Weigel et al., 1998; Liu et al., 2012).

### Ethical approval

The protocol of this study was approved by CUHAS/BMC joint ethics and scientific review committee with reference CREC/019/2014.

## Results

### Bacterial isolates

A total of 26 (13.3%) lactose-fermenting ESBL-producing bacteria were isolated from gut samples of 196 wild Nile tilapia fish from Lake Victoria. Diverse bacterial species were detected and included *C. braakii* (11/26, 42.3%), *E. cloacae* (5/26, 19.2%), *K. pneumoniae* (5/26, 19.2%), *E. coli* (4/26, 15.4%) and *Klebsiella oxytoca* (1/26, 3.9%). From 73 environmental samples 39 (53.4%) ESBL-producing enteric bacteria were isolated. Of these 39 isolates, 20 (51.3%) were *E. coli* and 19 (48.7%) were *K. pneumoniae*.

### Antimicrobial susceptibility testing

All isolates had MICs for cefotaxime and cefepime of >32 and >8 μg/ml respectively. High resistance rates to co-trimoxazole (*n* = 19, 73.1%), ciprofloxacin (*n* = 19, 73.1%), and gentamicin (*n* = 19, 73.1%) and tetracycline (*n* = 16, 61.5%) were observed among the 26 ESBL-producing isolates from fish. Environmental isolates were resistant to co-trimoxazole (*n* = 34, 87.2%), ciprofloxacin (*n* = 14, 38%), and gentamicin (*n* = 18, 46.1%). ESBL-producing isolates from fish were significantly more resistant to gentamicin and ciprofloxacin (*p* < 0.01: Table 1). In addition, all isolates were sensitive to meropenem and imipenem (MIC < 0.25 μg/ml), and all were sensitive to colistin.

**Table 1:** **Rate of resistance of ESBL-producing isolates from fish and environment to SXT, TET, CIP, and CN**.

**Antibiotic**	**ESBL fish (26)**	**ESBL environment (39)**	***P*-value**
SXT	19 (73%, 95% CI 55.9–90.6)	34 (87.2%, 95% CI 76–97)	0.0776
TET	17 (65.4%, 95% CI 47–83)	27(69.2%, 95% CI 54.7–83.6)	0.3741
CIP	19 (73%, 95% CI 55.9–90.6)	15 (38.5%, 95% CI 23–53)	0.0032
CN	19 (73%, 95% CI 55.9–90.6)	18 (46.1%, 95% CI 37–50	0.001

*SXT, sulphamethoxazole/trimethoprim; TET, tetracycline; CIP, ciprofloxacin, CN, gentamicin*.

### Analysis of antibiotic resistance genes

Each of the bacterial genome-sequenced fish and environmental isolates carried up to four different β-lactamase genes (ESBL, AmpC and other β-lactamases, Table 2). The most common ESBL gene was *bla*_CTX-M-15_, present both in fish (9/11) and environmental isolates (12/13). The non-ESBL β-lactamase gene *bla*_TEM-1_ was present in 7/11 fish isolates and 9/13 environmental isolates, whereas *bla*_OXA-1_ was present in 6/11 fish isolates and 1/13 environmental isolates. *K. pneumoniae* isolates from fish were found to harbor *bla*_SHV-11_ and *bla*_SHV-1_ due to the fact that *K. pneumoniae* chromosomally possesses *bla*_SHV-1/-11_. In addition, the fish isolates also harbored a number of AmpC type β-lactamase genes (*bla*_ACT-15_, *bla*_MIR-3_, *bla*_CMY-37_, *bla*_CMY-49_), which were not present in the environmental isolates. The latter two results might simply reflect the fact that while different types of enterobacterial species were isolated from fish, only *E. coli* were obtained from the environmental samples studied.

**Table 2:** **Antibiotic resistance phenotype and genotype of the sequenced isolates**.

**No**	**Species**	**Phenotypic resistance besides beta-lactams**	**Beta-Lactam Resistance genes**	**Trimethoprim resistance genes**	**Sulfonamide resistance genes**	**Aminoglycosides resistance genes**	**Quinolone resistance genes**	**Tetracycline resistance genes**
F006	*Enterobacter cloacae*		^*^bla_CTX-M-15_, *bla*_TEM-1B_, *bla*_ACT-15_, *bla*_OXA-1_	–	–	–	–	–
*F009*	*Enterobacter cloacae*	CIP, SXT, TET, CN	^*^bla_CTX-M-15_, *bla*_TEM-1B_, *bla*_ACT-15_, *bla*_OXA-1_	*dfrA14*	*sul1*	*aadA1, aac(6′)-lb-cr, aac(3)-lla*	*aac(6′)-lb-cr, qnrB1*	*tet(A)*
F016	*Enterobacter cloacae*		*bla*_MIR-3_	–	–	–	–	–
F017	*Enterobacter cloacae*	CIP, TET, CN	*bla*_TEM-1B_	–	*sul1*	*aadA1, aadA2*	–	*tet(A)*
F025	*Citrobacter braakii*	CIP, SXT, TET, CN	*bla*_CTX-M-15_, *bla*_OXA-1_, *bla*_TEM-1B_, *bla*_CMY-37_	*dfrA18*	*sul1, sul2*	*aac(3)-lla, aac(6′)-lb-cr, aadA2*	*aac(6′)-lb-cr, qnrB29*	*tet(D)*
F044	*Escherichia coli*	CIP, SXT, TET, CN	*bla*_CTX-M-15_, *bla*_TEM-1B_	*dfrA14*	*sul2*	*strB, strA*	*qnrS1*	*tet(A)*
F080	*Escherichia coli*	CIP	^*^bla_CTX-M-15_	–	–	–	*qnrB1*	–
F085	*Klebsiella pneumoniae*	CIP, SXT, TET, CN	^*^bla_CTX-M-15_, *bla*_OXA-1_, *bla*_SHV-11_	*dfrA14*	*sul2*	*acc(3)-lld, aac(6′)-lb-cr, strA, strB*	*aac(6′)-lb-cr, qnrB1, oqxA, oqxB*	*tet(D)*
F086	*Klebsiella pneumoniae*	CIP, SXT, TET, CN	^*^bla_CTX-M-15_, *bla*_OXA-1_, *bla*_SHV-11_	*dfrA14*	*sul2*	*acc(3)-lld, aac(6′)-lb-cr, strA, strB*	*aac(6′)-lb-cr, qnrB1, oqxA, oqxB*	*tet(D)*
F096	*Klebsiella pneumoniae*	CIP, SXT, TET, CN	*bla*_TEM-1B_, *bla*_CTX-M-15_, *bla*_SHV-1_	*dfrA30*	*sul2*	*aac(3)-lld*	*oqxA, oqxB*	–
F102	*Citrobacter braakii*	CIP, SXT, TET, CN	*bla*_CTX-M-15_, *bla*_CMY-49_, *bla*_TEM-1B_, *bla*_OXA-1_	*dfrA14*	*sul2*	*aac(3)-lla, aac(6′)-lb-cr, aadA1, strA, strB*	*aac(6′)-lb-cr, qnrB48*	–
SO005	*Escherichia coli*	SXT, TET, CN	*bla*_CTX-M-15_, *bla*_TEM-1B_	*dfrA14*	*sul2*	*strA, strB*	*qnrS1*	*tet(A)*
SO007	*Escherichia coli*	SXT, TET, CN	*bla*_CTX-M-15_, *bla*_TEM-1B_	*dfrA7*	*sul1, sul2*	*strA, strB*		*tet(A)*
SO008	*Escherichia coli*	CIP, SXT, TET, CN	*bla*_CTX-M-15_, *bla*_TEM-1B_	*dfrA14*	*sul2*	*strA, strB*	*qnrS1*	*tet(A)*
SO009	*Escherichia coli*	SXT, TET, CN	^*^bla_CTX-M-15_	*dfrA17*	*sul1*	*aadA5*		*tet(A)*
SO025	*Escherichia coli*	SXT, TET, CN	*bla*_CTX-M-15_	*dfrA14*	*sul2*	*strA, strB*	*qnrS1*	*tet(A)*
SO035	*Escherichia coli*	SXT, TET, CN	^*^bla_CTX-M-15_, *bla*_TEM-1B_	*dfrA1*	*sul2*	*aadA1, strA, strB*,		*tet(A)*
SO037	*Escherichia coli*	SXT, TET	*bla*_CTX-M-15_, *bla*_TEM-1B_	*dfrA14*	*sul2*	*strA, strB*	*qnrS1*	*tet(A)*
SO038	*Escherichia coli*	CIP	^*^bla_CTX-M-15_	–	–		*qnrB1*	–
SO042	*Escherichia coli*	SXT, TET, CN	*bla*_CTX-M-15_, *bla*_TEM-1B_	*dfrA17*	*sul1, sul2*	*aac(3)-IId,aadA5,strA, strB*	–	*tet(A)*
SO053	*Escherichia coli*	CIP, SXT, TET, CN	*bla*_CTX-M-55_, *bla*_TEM-1B_	*dfrA5*	*sul2*	*strA, strB*	–	*tet(A)*
SO060	*Escherichia coli*	CIP, SXT, TET, CN	*bla*_CTX-M-15_, *bla*_OXA-1_	*dfrA17*	*sul1, sul2*	*aac(6')-Ib-cr, aadA5, strA, strB*	*aac(6')-Ib-cr*	*tet(A)*
SO063	*Escherichia coli*	CIP, SXT, TET, CN	*bla*_CTX-M-15_, *bla*_TEM-1B_	*dfrA14*	*sul2*	*strA, strB*	*qnrS1*	*tet(A)*
SO069	*Escherichia coli*	SXT, TET	*bla*_CTX-M-15_, *bla*_TEM-1B_	*dfrA14*	*Sul1*	*aac(3)-IId,strA, strB*		*tet(A)*

* *in these isolates bla_CTX-M-15_ was located in the chromosome, CIP, ciprofloxacin; SXT, trimethoprim/sulphamethoxazole; CN, gentamicin; TET, tetracycline*.

Commonly occurring aminoglycoside resistance genes detected in both fish and environmental isolates were *aac(6*′*)-Ib-cr* (6/24), *str*A*/str*B (15/24), *aac(3)-IId* (5/24), and *aad*A1 (4/24) (Table 2). Other aminoglycoside resistance genes were present either only in fish isolates (*aad*A2, *aac(3)-IIa*) or in environmental samples (*aad*A5). Quinolone resistance genes were detected in 8/11 (72.7%) of fish isolates and in 7/13 (53.7%) of environmental isolates that were sequenced. Quinolone resistance genes detected in both populations comprised of the *aac(6*′*)-Ib-cr* (6/24), *qnr*B1 (5/24), and *qnr*S1 alleles. Other quinolone resistance genes were only present in fish isolates (*qnr*B29, *qnr*B48, *oqx*A, *oqx*B).

Of the 24 sequenced isolates, 20 (83.3%) harbored sulfonamide resistance genes (*sul1* or *sul2*), and 19 of these isolates harbored in addition a resistance gene encoding trimethoprim resistance (*dfr*A14, *n* = 11; *dfr*A17, *n* = 3; *dfr*A1, *dfr*A18, *dfr*A30, *dfr*A5, *dfr*A7, each *n* = 1). Tetracycline resistance genes, [*tet(A), n* = 15; *tet(D), n* = 3], were present in 18/24 isolates. Altogether, there was a direct correlation between resistance phenotype and resistance genotype in all 24 isolates.

Using *in silico* analysis *par*C mutations were detected in 7 *E. coli*, only 2 isolates had S80I mutation that is associated with fluoroquinolone resistance, while *gyr*A mutations were observed in 9 isolates (2 *E. cloacae* and 7 *E. coli*). S83 (S83T, S83L, and S83A) mutations that are associated with fluoroquinolone resistance were observed in 5/9 isolates. One isolate had both S83L and D87N.

### Plasmid-encoded heavy metal resistance operons and detergence genes

An analysis for the presence of plasmid-located heavy metal- and detergent- resistance genes revealed, that 8/24 isolates harbored the *qacEdelta* gene, conferring resistance to tertiary ammonium compounds. Seven (29.2%) of isolates sequenced carried a mercury resistance operon originally described in plasmid R478, and 4/24 (16.6%) isolates had genes mediating resistance to silver. Only one isolate carried genes involved in resistance to copper together with nickel/cobalt efflux system (Supplementary Table 2).

### Location of the *bla*_CTX-M_ gene and AmpC genes

Analysis of the whole genome sequences was used to identify and map the location of *bla*_CTX-M_ alleles. For 8/24 isolates, the *bla*_CTX-M-15_ gene was located in the chromosome. In two isolates *bla*_CTX-M-15_ was located on a phage-like plasmid, similar to the *E. coli* phage-like plasmid p1303_95 isolated from wound swab (isolate SO007, accession number of the reference: CP009168.1) and pECOH89 (isolate SO053, accession number of pECOH89: HG530657.1) (Falgenhauer et al., 2014). The isolate SO069 harbored *bla*_CTX-M-15_ on an IncI1 plasmid similar to pESBL-EA11 (Ahmed et al., 2012). Six of 24 sequenced isolates (one fish and five environmental isolates, F044, SO005, SO008, SO025, SO037, SO063, all *E. coli*) harbored a resistance cassette similar to the one present in plasmid pPGRT46 (accession number KM023153.1) that included both *qnrS*1 as well as *bla*_CTX-M-15_. The other isolates displayed *bla*_CTX-M-15_ or *bla*_CTX-M-55_ containing resistance cassettes with similarities to other ESBL-encoding plasmids (F025: pEC_L46, accession number GU371929.1; F102: pSTm-A54650, accession number LK056646.1; SO042: pSKLX3330, accession number KJ866866.1; SO060: pCA14, accession number CP009231.1). The isolates F016 and F017 did not harbor any ESBL resistance gene. It should be noted that because conjugation experiments were not done plasmid location could only be suggested.

**Figure 2:**
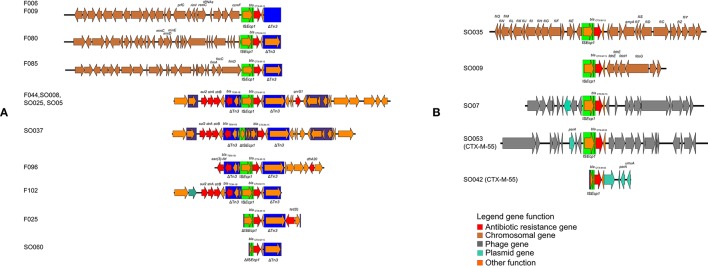
**Schematic depiction of the genetical environment of ***bla***_**CTX-M**_ genes in the ***bla***_**CTX-M**_-encoding isolates**. **(A)** Genetical environments associated with Tn*3* transposon deletion, **(B)** gentical environments not associated with Tn*3* transposon deletion.

In 13 of 22 ESBL-encoding isolates, the environment of the ESBL gene was characterized by a Tn*3* transposon deletion in the vicinity of the *bla*_CTX-M_ gene (Figure 2). This included four isolates harboring a chromosomally located *bla*_CTX-M-15_ allele. The other isolates, including two chromosomally located *bla*_CTX-M-15_ and the single *bla*_CTX-M-55_ isolate, did not have any Tn*3*-related sequences. All ampC genes detected were located in the chromosomes (F006, F009, F016, F025, F102).

### Multi locus sequence types and whole genome phylogeny

**Figure 3:**
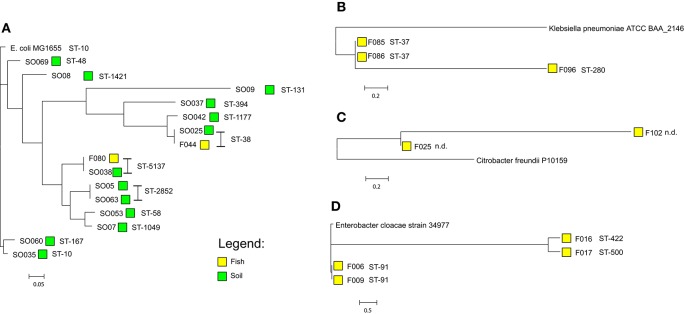
**Core-genome based phylogeny of the sequenced isolates according to their species (A)** *Escherichia coli* (reference *E. coli* MG1655, accession number U00096.3), **(B)** *Klebsiella pneumoniae* (reference *Klebsiella pneumoniae* strain ATCC BAA-2146, accession number CP006659), **(C)** *Citrobacter braakii* (using *Citrobacter freundii* P10159, accession number CP012554.1 as a reference as no complete genome of *Citrobacter braakii* is presently available), **(D)** *Enterobacter cloacae (E. cloacae* strain 34977, accession number CP010376). The phylogenetic analysis was performed using Harvest Suite.

*E. coli* isolates sequence type (ST) ST-38 and ST-5173 were present in both fish and environmental isolates. Among the 13 *E. coli* environmental isolates 12 different STs were observed. The fifteen *E. coli* isolates, 13 from the environment and 2 from fish, grouped into five clusters using genome-based phylogenetic analysis. The tree was generated on the basis of a core genome accounting for 78% (3.62 Mbp) of the reference genome (*E. coli* MG1655). The two *E. coli* isolates from fish have 100% similarity with respective ST isolates derived from the environment (SO025 and F044, ST-38; F080 and SO038, ST-5137; Figure 3). *E. cloacae* isolates from fish displayed three different STs (ST-91 *n* = 2; ST-422 *n* = 1; ST-500 *n* = 1), based on an analysis of core genome content of around 68% (3.33 Mbp) using *E. cloacae* strain 34977 as reference. The two ST-91 isolates were more closely related to each other, than the other two isolates. *K. pneumoniae* isolates were typed as ST-37 (2/3) with a single isolate as ST-280 (1/3). The core genome with *K. pneumoniae* strain ATCC BAA-2146 (accession number CP006659) as reference accounted for 88% (4.78 Mbp) of its genome size. The ST-37 and ST-280 are quite different from one other. The *C. braakii* isolates could not be assigned to any ST, because there is no typing scheme available. Based on 82% (4.17 Mbp) core genome size using *C. freundii* strain P10159 as a reference genome, these two isolates are very distinct from each other (Figure 3). *E. coli* were grouped in different phylogroups based on scheme of Clermont et al. (2000). A total of 7/15(46%) of *E. coli* isolates belong to the newly described phylogroups E (Table 3).

**Table 3:** **Baseline characteristics of the sequenced isolates**.

**No**	**Species**	**Sequence type**	**Phylogroups**	**Source**
F006	*Enterobacter cloacae*	ST-91		Fish
F009	*Enterobacter cloacae*	ST-91		Fish
F016	*Enterobacter cloacae*	ST-422		Fish
F017	*Enterobacter cloacae*	ST 500		Fish
F025	*Citrobacter braakii*	^*^		Fish
F044	*Escherichia coli*	ST-38	E	Fish
F080	*Escherichia coli*	ST-5173	B1	Fish
F085	*Klebsiella pneumoniae*	ST-37		Fish
F086	*Klebsiella pneumoniae*	ST-37		Fish
F096	*Klebsiella pneumoniae*	ST-280		Fish
F102	*Citrobacter braakii*	^*^		Fish
SO005	*Escherichia coli*	ST-2852	B1	Environment
SO007	*Escherichia coli*	ST-1049	B1	Environment
SO008	*Escherichia coli*	ST-1421	A	Environment
SO009	*Escherichia coli*	ST-131	B2	Environment
SO025	*Escherichia coli*	ST-38	E	Environment
SO035	*Escherichia coli*	ST-10	E	Environment
SO037	*Escherichia coli*	ST-394	E	Environment
SO038	*Escherichia coli*	ST-5173	B1	Environment
SO042	*Escherichia coli*	ST-1177	E	Environment
SO053	*Escherichia coli*	ST-58	B1	Environment
SO060	*Escherichia coli*	ST-167	E	Environment
SO063	*Escherichia coli*	ST-2852	B1	Environment
SO069	*Escherichia coli*	ST-48	E	Environment

* *There is no MLST scheme available for Citrobacter braakii*.

### Plasmid replicon types

**Figure 4:**
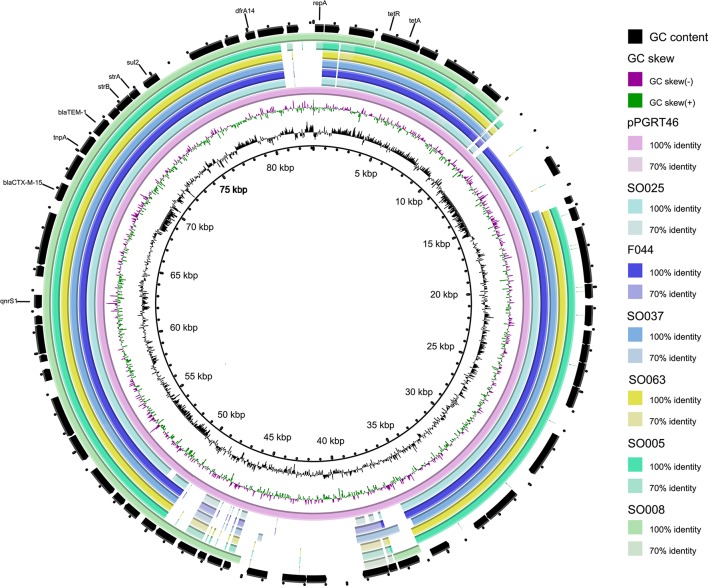
**Comparative analysis of the whole genome sequences with plasmid pPGRT46**. Alignments were performed using BRIG and depict percent identity between genes from the draft sequences of various plasmids described in this study and the reference plasmid.

Of the eleven fish isolates tested, seven (63.6%) were found to carry IncF plasmid replicon types as compared to 5/13 (38.5%) of environmental isolates (Table 4). The IncF pMLST types detected in fish isolates were F-: A-:B36, K4:A-:B- and K5:A-:B-. For the environmental isolates IncF pMLST types detected were F2: A-:B-, F29:-A-:B10 and F31:A4:B1. The IncI1 plasmids detected in *E. coli* from environment were classified using pMLST either as ST-31 and unknown pMLST while that in *E. coli* from fish had an unknown IncI1 pMLST. Two out of four *E. cloacae* isolates from fish harbored IncF plasmids with identical pMLST (F-:A-:B36). We extracted contig sequences of the F044, SO005, SO008, SO025, SO037, SO063, carrying the resistance cassette present in pPGRT46 (Fortini et al., 2015) (accession no. KM023153) and compared them to this plasmid to examine for overall homology. These isolates showed an overall overlap of between 76 and 90% in their nucleotide sequences with plasmid pPGRT46 (Figure 4).

**Table 4:** **Plasmid characteristics of the sequenced isolates, n.t.: not typable using IncHI1 pMLST scheme (no pMLST alleles present); # variants with homology or coverage less than 100%**.

**No**	**Species**	**Plasmid Replicon type**	**pMLST**	**ESBL gene**	**accession number of best reference**	**location**
F006	*Enterobacter cloacae*	IncFII, IncFIB	F-:A-:B36#	*bla*_CTX-M-15_	CP011650.1	Chromosome
F009	*Enterobacter cloacae*	IncFII, IncFIB	F-:A-:B36#	*bla*_CTX-M-15_	CP011650.1	Chromosome
F016	*Enterobacter cloacae*	IncFIB(K)	F-:A-:B-	no ESBL gene	/	/
F017	*Enterobacter cloacae*	IncFII, IncR	F-:A-:B-	no ESBL gene	/	/
F025	*Citrobacter braakii*	No replicon		*bla*_CTX-M-15_	GU371929.1	Plasmid
F044	*Escherichia coli*	IncI1, IncY	I1: new	*bla*_CTX-M-15_	KM023153.1	Plasmid
F080	*Escherichia coli*	No replicon	–	*bla*_CTX-M-15_	CP011018.1	Chromosome
F085	*Klebsiella pneumoniae*	IncFII, IncFIB(K), IncHI1B	F: K4:A-:B-, IncHI1: n.t.	*bla*_CTX-M-15_	CP006659.2	Chromosome
F086	*Klebsiella pneumoniae*	IncFII, IncFIB(K), IncHI1B	F: K4:A-:B-, IncHI1: n.t.	*bla*_CTX-M-15_	CP006659.2	Chromosome
F096	*Klebsiella pneumoniae*	IncFII(K), IncR	K5:A-:B-	*bla*_CTX-M-15_	KM023153.1	Plasmid
F102	*Citrobacter braakii*	No replicon	–	*bla*_CTX-M-15_	LK056646.1	Plasmid
SO005	*Escherichia coli*	IncY	–	*bla*_CTX-M-15_	KM023153.1	Plasmid
SO007	*Escherichia coli*	IncI1, IncP, IncY	I1: new	*bla*_CTX-M-15_	CP009168.1	Plasmid
SO008	*Escherichia coli*	No replicon	–	*bla*_CTX-M-15_	KM023153.1	Plasmid
SO009	*Escherichia coli*	IncFII, IncFIA	F2:A1:B-	*bla*_CTX-M-15_	CP013658.1	Chromosome
SO025	*Escherichia coli*	IncY	–	*bla*_CTX-M-15_	KM023153.1	Plasmid
SO035	*Escherichia coli*	No replicon	–	*bla*_CTX-M-15_	LM995868.1	Chromosome
SO037	*Escherichia coli*	IncFII, IncY	–	*bla*_CTX-M-15_	KM023153.1	Plasmid
SO038	*Escherichia coli*	No replicon	–	*bla*_CTX-M-15_	CP011018.1	Chromosome
SO042	*Escherichia coli*	IncFII, IncFIB	F29:A-:B10	*bla*_CTX-M-55_	KJ866866.1	Plasmid
SO053	*Escherichia coli*	IncFII, IncFIB, IncQ1	F2:A-:B1	*bla*_CTX-M-15_	HG530657.1	Plasmid
SO060	*Escherichia coli*	IncFII, IncFIA, IncFIB, IncFII	F31#:A4:B1	*bla*_CTX-M-15_	CP009231.1	Plasmid
SO063	*Escherichia coli*	IncY	–	*bla*_CTX-M-15_	KM023153.1	Plasmid
SO069	*Escherichia coli*	IncI1	I1: ST-31	*bla*_CTX-M-15_	CP003290.1	Plasmid

## Discussion

Lake Victoria is Africa's largest lake by area, and borders three East-African countries: Tanzania (49%), Uganda (45%), and Kenya (6%). It is not only the major source of fish consumed by Mwanza residents but also receives biologically treated wastewater effluent from Mwanza city, where a tertiary hospital serving eight regions in Tanzania is located. Studies performed in this hospital have shown high rates of ESBL-producing *K. pneumoniae* and *E. coli* carrying *bla*_CTX-M-15_ in IncF plasmids (Mshana et al., 2011, 2013). In addition recent studies involving isolates from animals and humans from community have shown the *bla*_CTX-M-15_ allele to be predominant (Mshana et al., 2016; Seni et al., 2016).

In the present study, a significant proportion of fish gut and environmental samples were positive for ESBL-producing bacteria. As previously observed in isolates from humans in hospitals (Mshana et al., 2011, 2013), the majority of these isolates were multiply resistant to co-trimoxazole, gentamicin, tetracycline, and ciprofloxacin. In addition, it was observed that ESBL isolates from fish were significantly more resistant to ciprofloxacin and gentamicin. This could be explained by the fact majority of the environment isolates were *E. coli* while other genera such as Citrobacter and Enterobacter which tend to be resistant to ciprofloxacin and gentamicin (Dos Santos et al., 2015) formed majority of isolates from Fish. Such high AMR rates detected in the isolates from gut contents of wild fish are of concern and indicate strong anthropogenic environmental contamination of the Lake Victoria. The presence of ESBL isolates in the environmental samples suggests that the Lake Victoria is being contaminated by sewage from the hospitals and from animals (Mshana et al., 2009; Kayange et al., 2010; Moremi et al., 2014; Seni et al., 2016). Farming activities that use of animal manure together with agricultural waste from the community generate steady contaminated effluents along the borders of Lake Victoria. Climatic conditions including sustained periods of rain or man-made erosion may further contribute to increasing the presence of these bacteria in the lake.

The prevalence of ESBL-producing *E. coli* from wild fish was 2% (4/196) which is comparable to the results previously reported from China (Jiang et al., 2012) but lower than in Egypt which reported a prevalence of 4% (Ishida et al., 2010). Unlike the two studies from Egypt and China which were conducted among farmed fish with antibiotic exposure, the investigated isolates here were from uncultured free-living fish and emphasize the role of environmental pollution with AMR isolates as previously published (Kümmerer, 2009; Guenther et al., 2011).

As was already noted in studies from the Asian, European and the African continents which documented the emergence and spread of *bla*_CTX-M-15_ in humans and cattle (Madec et al., 2012; Zhang et al., 2013; Rafaï et al., 2015), CTX-M-15 was also found to be the predominant allele in this study. The gene was located on the chromosome or on different plasmids indicating extreme environmental mobility. The *bla*_CTX-M-15_ allele has been reported in other studies (Dobiasova et al., 2015; Fortini et al., 2015; Ibrahim et al., 2015) of Gram-negative bacterial isolates from humans, fish, and animals, suggesting a transmission/circulation of this gene among different settings.

Two plasmid-mediated AmpC enzymes were detected in this study from fish isolates: *bla*_MIR-1_ and *bla*_ACT-15_, this is in contrast with a previous report that showed the presence of plasmid-mediated *bla*_CMY-2_ allele in fish (Welch et al., 2009). No isolates carrying *bla*_CMY-2_ were detected in this study. However, a chromosomally located *bla*_CMY-37_ which had previously been described in *C. freundii*, was detected in a *C. braakii*, (Ahmed and Shimamoto, 2008). One resistant isolate harbored neither a plasmid-based AmpC nor a detectable ESBL enzyme (F017) suggesting the resistance observed might be due to unknown mechanisms or the presence of resistance genes in the chromosome. The chromosomally located genes cannot be detected using the software from the Center for Genomic Epidemiology because the database targets for transferable resistance genes only. As in previous studies performed in China and Egypt among farmed fish (Ishida et al., 2010; Jiang et al., 2012) plasmid-mediated quinolone resistance genes such as *aac(6*′*)-Ib-cr, qnr*B and *qnr*S were also detected in this study in both sampled populations.

*E. coli* ST-38 which was also observed in humans in hospitals, humans in the community and in livestock/ companion/domestic animals in Mwanza city (Mshana et al., 2011, 2016; Seni et al., 2016) was detected in this study in both fish and environmental samples. According to the new phylogroup scheme by Clermont et al. (2000), all these Isolates (human, livestock/companion animal, fish/environment) were grouped as the new phylogroup E emphasizing the uniformity of these strains in our setting. The ST-38 *E. coli* from fish and the environment as well as other ST types from environment harbor resistance cassettes that include CTX-M-15 and QnrS1. A similar resistance cassette was previously detected on an IncY plasmid detected in isolates from healthy pregnant women in Nigeria, and from ESBL-producing isolates from livestock and companion animal isolates in the Mwanza region (Fortini et al., 2015; Seni et al., 2016). The sequence overlap with the plasmid pPGRT46 was very high (up to 90%). These findings draw attention to the likelihood that this resistance cassette and its plasmid have spread centrally across the African continent into multiple *E. coli* genotypes and in different environments.

The *E. coli* ST-38 was observed in both fish and environment isolates as documented above was previously detected in humans and animals in the same setting. This finding is worrisome as zoonotic transmission is possible and its association with human colonization and infection might pose treatment challenges to human health. The transmission and persistence of ESBL-producing bacteria through the food chain and environment through sewage contamination has been documented previously (Beninati et al., 2015; van Hoek et al., 2015). The findings of this study suggest the possibility of the transmission of both ESBL-producing clones and plasmids between humans and wild fish through environmental contamination and indicate that anthropogenic activities and the food chain as potential factors for the persistence and dissemination of *bla*_CTX-M-15_ in Mwanza City. The current study did not observed *E. coli* ST-131 from fish however it was obtained from environment; this could be due to that only 2 out 4 isolates from fish were sequenced. Further studies with large number of *E. coli* from fish are needed to confirm the role of food chain in the persistence of *bla*_CTX-M-15_ in Mwanza city.

## Conclusion

This is the first report on the epidemiology of ESBL-producing Gram-negative bacteria from fish in Lake Victoria and its surrounding environment. More than 70% of the sequenced bacterial isolates carried quinolone and aminoglycoside resistance genes. Detection of isolates/plasmids carrying *bla*_CTX-M-15_ which have been also found in humans and companion/livestock animals in the same region suggest environmental contamination with sewage effluents from humans an animal sources. Our data suggest that additional efforts to implement better sanitation and control sewage management are warranted. Many common illnesses in particular diarrhea, can be attributed to poor sanitation and unsafe water (Cheng et al., 2005) which exacerbates antibiotic use and contributes significantly to the problem of antibiotic resistance. Policy measures to improve water and sanitation quality could greatly contribute to the reduction of ESBL-resistance in this region.

## Author contributions

NM, MM, CI, TC, and SM conceived, designed and executed the study; EM, NM, and SM collected the data and samples; NM, EM, and SM performed laboratory analysis; LF and TC performed WGS; LF, HG, TC, and SM analyzed the data; NM, LF, TC, and SM wrote the manuscript which was critically reviewed by all authors. All authors have read and approved the final draft of the manuscript.

### Conflict of interest statement

The authors declare that the research was conducted in the absence of any commercial or financial relationships that could be construed as a potential conflict of interest.
