# Supplementary material for: Predominance of CTX-M-15 among ESBL Producers from Environment and Fish Gut from the Shores of Lake Victoria in Mwanza, Tanzania
Source: Front Microbiol. 2016 Dec 1;7:1862. doi: 10.3389/fmicb.2016.01862 (PMC5130978; doi:10.3389/fmicb.2016.01862)
Supplement: Supplementary file 1 [file Table1.DOCX]

**Supplementary Table 1: References for the assessment of the plasmid-located heavy metal and detergent resistance**

| Type of resistance | Genes involved | Reference plasmid | Reference number | position | size |
| --- | --- | --- | --- | --- | --- |
| copper resistance | *copE2ABCDRSE1* | R478 | NC_005211.1 | 139283-146818 | 7536 bp |
| tellurite | *terY3Y2XY1WZABCDEF* | R478 | NC_005211.1 | 64400-81187 | 16788 bp |
| silver | *silPABCRSE* | R478 | NC_005211.1 | 125052-137985 | 12934 bp |
| arsenate | *arsHRBC* | R478 | NC_005211.1 | 159291-162175 | 2885 bp |
| Nickel/cobalt efflux system | *rcnRA* | pRH-R27 | LN555650 | 193693-195203 | 1511 bp |
| mercury | *merRTPCADE* | R478 | NC_005211.1 | 106086-110062 | 3977 bp |
| detergence | *qacEdelta* | pRH-R27 | LN555650 | 259227..259574 | 348 bp |
