# Supplementary material for: Predominance of CTX-M-15 among ESBL Producers from Environment and Fish Gut from the Shores of Lake Victoria in Mwanza, Tanzania
Source: Front Microbiol. 2016 Dec 1;7:1862. doi: 10.3389/fmicb.2016.01862 (PMC5130978; doi:10.3389/fmicb.2016.01862)
Supplement: Supplementary file 2 [file Table2.DOCX]

**Supplementary Table 2: Presence/absence of plasmid-encoded heavy metal /detergent resistance genes (coverage and percentage identity higher than 80%).**

| **NO** | **Species** | copper | tellurite | silver | Arsenate | Nickel/cobalt efflux system | mercury | detergence |
| --- | --- | --- | --- | --- | --- | --- | --- | --- |
| F006 | *Enterobacter cloacae* |  |  | *+* |  |  | *+* | *+* |
| F009 | *Enterobacter cloacae* |  |  | *+* |  |  | *+* | *+* |
| F016 | *Enterobacter cloacae* | *+* |  | *+* |  |  |  |  |
| F017 | *Enterobacter cloacae* |  |  |  |  |  | *+* | *+* |
| F025 | *Citrobacter braakii* |  |  |  |  |  |  | *+* |
| F044 | *Escherichia coli* |  |  |  |  |  |  |  |
| F080 | *Escherichia coli* |  |  |  |  |  |  |  |
| F085 | *Klebsiella pneumoniae* |  |  |  |  |  | *+* |  |
| F086 | *Klebsiella pneumoniae* |  |  |  |  |  | *+* |  |
| F096 | *Klebsiella pneumoniae* |  |  |  |  |  |  |  |
| F102 | *Citrobacter braakii* | *+* |  | *+* |  | *+* |  |  |
| SO005 | *Escherichia coli* |  |  |  |  |  |  |  |
| SO007 | *Escherichia coli* |  |  |  |  |  | *+* | *+* |
| SO008 | *Escherichia coli* |  |  |  |  |  |  |  |
| SO009 | *Escherichia coli* |  |  |  |  |  |  | *+* |
| SO025 | *Escherichia coli* |  |  |  |  |  |  |  |
| SO035 | *Escherichia coli* |  |  |  |  |  |  |  |
| SO037 | *Escherichia coli* |  |  |  |  |  |  |  |
| SO038 | *Escherichia coli* |  |  |  |  |  |  |  |
| SO042 | *Escherichia coli* |  |  |  |  |  |  | *+* |
| SO053 | *Escherichia coli* |  |  |  |  |  | *+* |  |
| SO060 | *Escherichia coli* |  |  |  |  |  |  | *+* |
| SO063 | *Escherichia coli* |  |  |  |  |  |  |  |
| SO069 | *Escherichia coli* |  |  |  |  |  |  |  |
